# Deep Learning Models for Automatic Classification of Anatomic Location in Abdominopelvic Digital Subtraction Angiography

**DOI:** 10.1007/s10278-024-01351-z

**Published:** 2025-01-09

**Authors:** Reza Moein Taghavi, Amol Shah, Vladimir Filkov, Roger Eric Goldman

**Affiliations:** 1 Department of Radiology, UC Davis School of Medicine, University of California, Davis, 4860 Y Street, Suite 3100, Sacramento, CA 95817-2307 USA; 2 Department of Computer Science, University of California, Davis, Davis, CA USA

**Keywords:** Deep learning, Digital subtraction angiography, Interventional radiology, Anatomic localization

## Abstract

**Purpose:**

To explore the information in routine digital subtraction angiography (DSA) and evaluate deep learning algorithms for automated identification of anatomic location in DSA sequences.

**Methods:**

DSA of the abdominal aorta, celiac, superior mesenteric, inferior mesenteric, and bilateral external iliac arteries was labeled with the anatomic location from retrospectively collected endovascular procedures performed between 2010 and 2020 at a tertiary care medical center. “Key” images within each sequence demonstrating the parent vessel and the first bifurcation were additionally labeled. Mode models aggregating single image predictions, trained with the full or “key” datasets, and a multiple instance learning (MIL) model were developed for location classification of the DSA sequences. Model performance was evaluated with a primary endpoint of multiclass classification accuracy and compared by McNemar’s test.

**Results:**

A total of 819 unique angiographic sequences from 205 patients and 276 procedures were included in the training, validation, and testing data and split into partitions at the patient level to preclude data leakage. The data demonstrate substantial information sparsity as a minority of the images were designated as “key” with sufficient information for localization by a domain expert. A Mode model, trained and tested with “key” images, demonstrated an overall multiclass classification accuracy of 0.975 (95% CI 0.941–1). A MIL model, trained and tested with all data, demonstrated an overall multiclass classification accuracy of 0.966 (95% CI 0.932–0.992). Both the Mode model with “key” images (*p* < 0.001) and MIL model (*p* < 0.001) significantly outperformed a Mode model trained and tested with the full dataset. The MIL model additionally automatically identified a set of top-5 images with an average overlap of 92.5% to manually labelled “key” images.

**Conclusion:**

Deep learning algorithms can identify anatomic locations in abdominopelvic DSA with high fidelity using manual or automatic methods to manage information sparsity.

**Supplementary Information:**

The online version contains supplementary material available at 10.1007/s10278-024-01351-z.

## Introduction

Anatomic localization is a critical component of angiographic interpretation during and after minimally invasive image-guided endovascular procedures. Digital subtraction angiography (DSA) remains the gold-standard imaging modality for anatomic localization, mapping of anatomy, and diagnosis of vascular pathology during image-guided procedures in the abdomen and pelvis. Identifying the vascular structures opacified during a DSA provides the requisite localization and is required for appropriate interpretation of the imaging findings, intraprocedural guidance, and post-procedural reporting and billing.

While investigation toward automatic interpretation in the abdominopelvic angiography is limited, significant literature has demonstrated the feasibility and efficacy of deep learning algorithms for multiple medical imaging modalities. In the field of angiography, deep learning algorithms have demonstrated impressive results for diagnosis and segmentation in cross-sectional three-dimensional CT and MR angiography [1–3]. Vessel segmentation of two-dimensional time-series fluoroscopic angiography has been demonstrated in the coronary vessels [4–6]. Localization of arterial structures on DSA obtained during routine cardiology clinical practice, including non-coronary vessels such as the femoral artery and aorta, has recently been demonstrated as a component of an end-to-end pipeline for coronary stenosis identification [7].

The challenges of automated interpretation in abdominopelvic DSA are multifaceted. The diversity of vascular anatomy and varying pathophysiology contributes substantially to the interpretation challenge. Motion artifact is particularly pronounced in abdominopelvic DSA due to diaphragmatic excursion associated with breathing, bowel peristalsis, and renal contrast excretion. Differences in imaging acquisition parameters such as patient or equipment orientation, image magnification, contrast dose, and contrast infusion rate contribute to image variability. The transient nature of angiographic findings due to physiologic and pathologic vascular flow of contrast limits the useful frames of an angiographic sequence, creating sparsity within the data and adding to the interpretive challenge.

Anatomic localization is an essential initial step for accurate human, or algorithmic, interpretation. Interpretation of a DSA sequence is predicated on knowledge of the anatomy as normal physiologic findings in one vascular territory may be pathologic in another. We hypothesize that deep learning algorithms can accurately identify the anatomic location of an angiographic sequence, and manual and algorithmic mechanisms can mitigate the problem of data sparsity. The purpose of this study is to explore the information available for anatomic localization in abdominopelvic DSA sequences acquired during routine endovascular intervention at a tertiary care medical center and to evaluate deep learning methods for this localization using manual and automatic methods to manage data sparsity.

## Materials and Methods

### Data Collection, Curation, and Labeling

A retrospective review of the institutional PACS database identified endovascular procedures performed between 2010 and 2020. Inclusion criteria were patient age greater than or equal to 18 years at the time of the endovascular procedure, angiographic studies of the abdominopelvic arterial structures, and a cross-sectional study in the PACS within 150 days of the index angiographic study. Patients with post-surgical vascular anatomy due to visceral organ transplant were excluded.

**Fig. 1:**
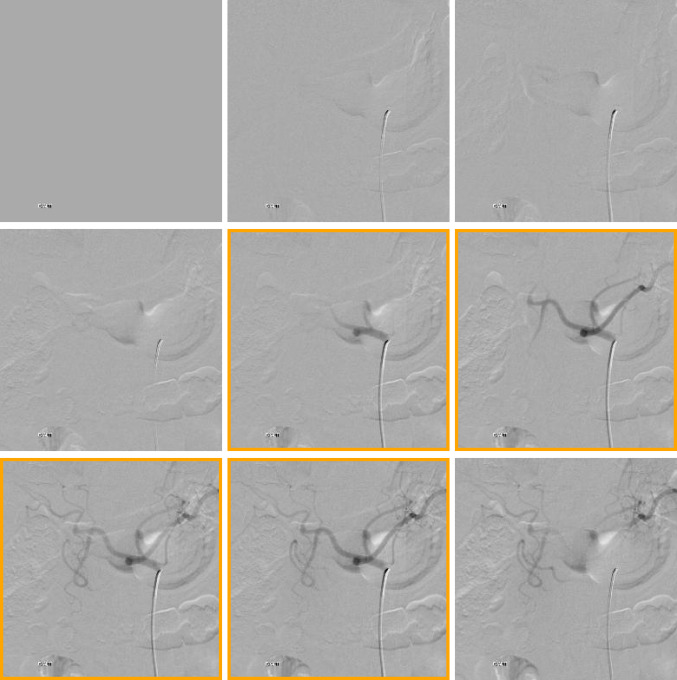
“Key” image labeling. A 9-image sequence with “key” images framed in gold. All images in an angiographic sequence in which the parent vessel and first bifurcation and opacified are labeled “key”

Individual DSA sequences from each study were selected for inclusion in the experimental dataset. The requirement for cross-sectional imaging was specified to allow direct comparison of the conventional angiography for more accurate anatomic location labeling. These CT and MRI images were not included in the experimental data. Manual labeling of the DSA sequences was performed separately by two interventional radiology attending physicians (A.S., R.G.) with 4 and 5 years of post-fellowship experience. Individual DSA series representing the abdominal aorta, celiac trunk, superior mesenteric artery (SMA), inferior mesenteric artery (IMA), right external iliac artery (EIA), and left EIA were selected and labeled from the identified studies. Unsubtracted angiography sequences and sequences acquired from other anatomic locations were excluded. Individual images in each DSA series were additionally labeled as “key” or non- “key.” “Key” images were defined as the set of sequential images in which the parent vessel and first bifurcation were opacified (Fig. 1). The remainder of the images in the sequence was labeled as non- “key.” Discrepancies between the labels were resolved subsequently by consensus. Interobserver agreement of the labeling was assessed using Cohen’s kappa coefficient for the classification of the angiographic location, and separately for the key images, without weighting either of the domain expert labelers.

We formed our modeling dataset by randomly selecting and labeling a cohort of 230 procedures from 160 unique patients. Based on a class analysis demonstrating a substantial imbalance in the IMA class, these data were supplemented with an additional 46 procedures from 45 patients curated to balance the label classes. The additional studies were identified for inclusion through a search of the associated procedure report for the text phrase “inferior mesenteric.”

The angiographic sequences were split randomly into training (70%), validation (15%), and testing (15%) partitions, each containing non-overlapping patients. A split with the most even class balance across the training, validation, and testing partitions was chosen through an automated search of 9e6 random permutations of the patients and procedures (see Supplemental Materials for details).

**Fig. 2:**
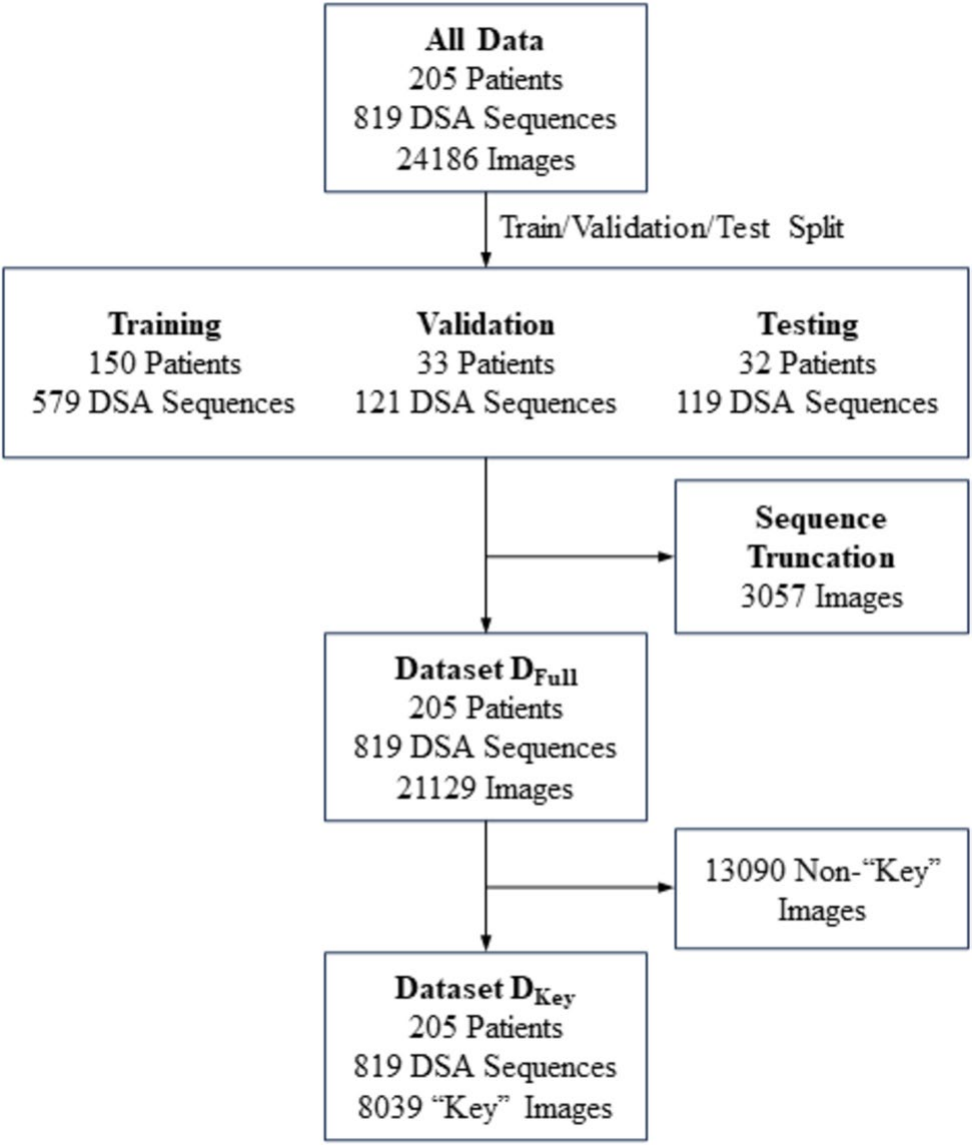
Datasets for training, validation, and testing. The angiographic sequences were split randomly into training (70%), validation (15%), and test (15%) sets, each containing non-overlapping patients. Sequences with more than 50 images were truncated at 50 images. Dataset D_Full_ is comprised of all individual images from the 205 unique patients and 276 procedures after splitting into training, validation, and test sets and sequence truncation. Dataset D_Key_ is a subset of D_Full_ comprised of the “key” images only for each sequence

Two related datasets were constructed to develop and evaluate deep learning models for location classification (Fig. 2). Dataset D_Full_ contains all images from each angiographic sequence, including both “key” and non- “key” labeled images. Dataset D_Key_, a subset of D_Full_, contains only the images labeled as “key.” All images from a given sequence contain the same anatomic location label.

### Technical Details

All image processing, model development, training, and testing were performed on a machine with an Ubuntu 20.04.5 operating system, an Intel Xeon Gold 6226R CPU, and an NVIDIA A100 graphical processing unit with CUDA 11.2 and cuDNN 8.4. Code was written in Python 3.9.12, leveraging the MONAI 1.2.0, Pandas 1.4.3, and PyTorch 2.0.1 packages.

### Image and Sequence Pre-processing

Image pre-processing was performed using the Matplotlib 3.5.2 and Open CV 4.7.0.72 packages. To reduce GPU memory requirements and match the input specification in the downstream deep learning analyses, the DICOM image data, originally with a 16-bit pixel depth and 1024 × 1024 matrix size, were resized to a 256 × 256 matrix size for each individual image in a DSA sequence and saved in the default JPEG format with an 8-bit pixel depth. Sequences exceeding 50 images were capped at 50 for computational efficiency and consistency across deep learning models. The absence of patient identifying information was confirmed through manual review.

### Model Architectures and Development

**Fig. 3:**
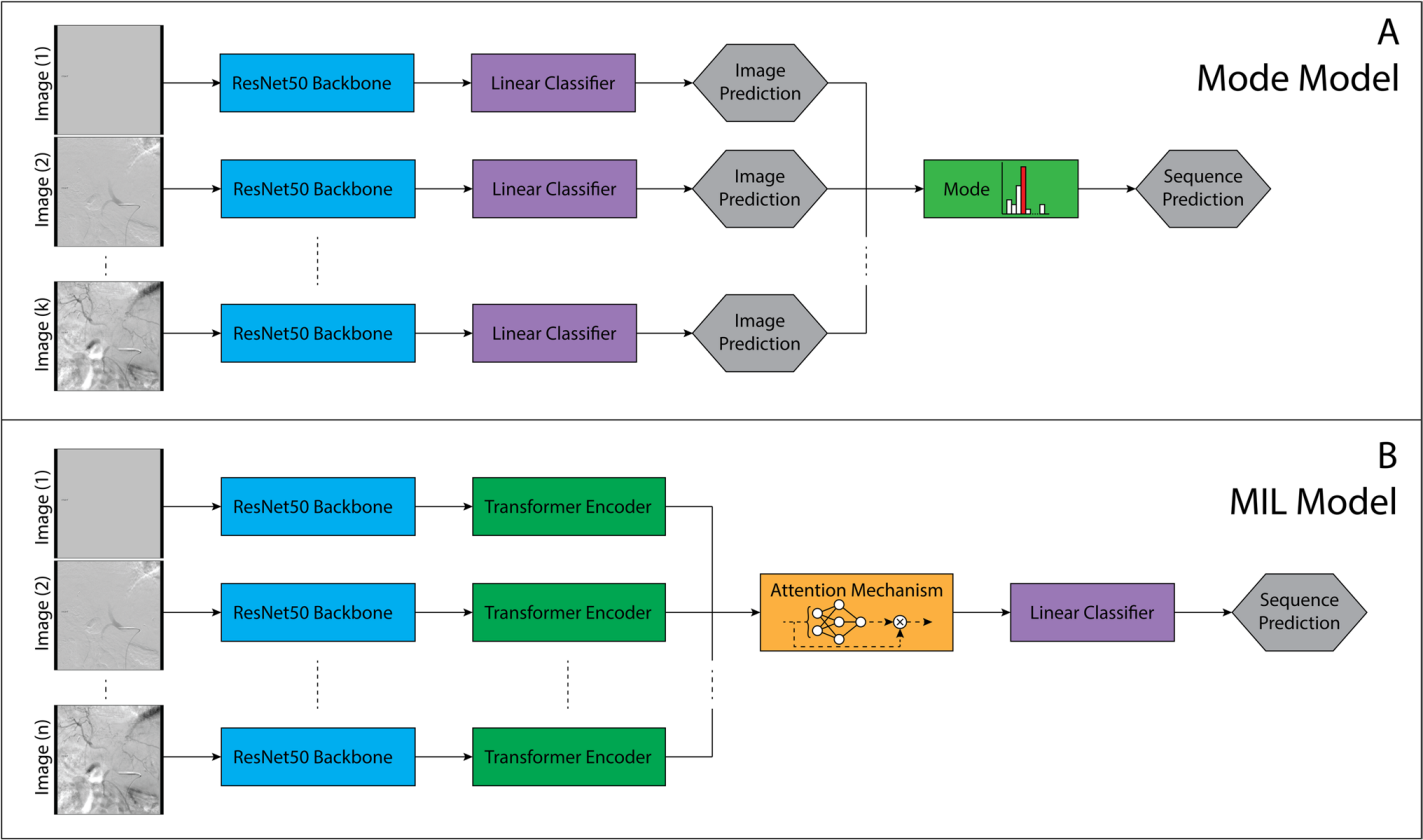
Schematic representations of the deep learning model architectures. **A** Mode Model. The angiographic location of individual images is predicted using a ResNet50 convolutional neural network (CNN) and fully connected linear classifier. The model accepts a DSA sequence of an arbitrary number of k images. The prediction for the DSA sequence is calculated by the mode of the individual predictions. **B** MIL Model. The extracted features of images from ResNet50 CNN are supplied to a transformer encoder and subsequently an attention mechanism. The model accepts a sequence of *n* images, where for sequences < *n*, the sequence is padded with blank images. The model predicts the anatomic location of the sequence and the “key” diagnostic images within the sequence based upon the attention weights

Two distinct classification architectures were developed to localize the angiographic anatomy of DSA sequences. The first architecture aggregates individual image predictions, and models utilizing this architecture will be henceforth referred to as Mode models. The second architecture utilizes a multiple instance learning (MIL) framework and will be referred through the remainder of the text as a MIL model. Schematic representations of two model architectures are provided in Fig. 3.

#### Mode Models

Classification models to predict the anatomic location of individual angiographic images were constructed. To generate a prediction of the anatomic location of the entire DSA sequence, a mode operation was performed across the combined individual image predictions [7]. Two distinct mode models were developed. Mode Model_Full_ utilized training and validation partitions of the entire data, D_Full_. Similarly, Mode Model_Key_ utilized only “key” images contained in Dataset D_Key_.

The base models for individual image classification, Fig. 3A, utilize the ResNet50 convolutional neural network (CNN) [8] which has demonstrated favorable performance in medical image classification applications. Experimentation was performed with alternate model architectures including VGG-16 [9], Inception-V3 [10], Swin-Transformer [11], and EfficientNet-B7 [12] and demonstrated no substantial performance improvement over ResNet50. The training approach, data augmentation strategies, and hyperparameters are included in the Supplemental Materials.

#### Multiple Instance Learning Model

A deep multiclass MIL model was developed to identify the anatomic location and provide a weighted score of the contribution of each individual image to the classification based on an attention mechanism [13]. The challenges of angiographic sequence classification resembles multiple weakly supervised tasks solved with MIL architectures and closely parallels whole slide image (WSI) pathology classification [14–16]. As the size of WSI limits computation on the entire image, algorithmic approaches frequently divide the image into smaller computationally manageable image patches. Noting the pathologic diagnosis may depend on patterns in spatially separated patches, algorithms must account for patch dependencies. In DSA sequences, a single image is often insufficient to infer the anatomic location. WSI patch dependencies are, therefore, analogous to the anatomic information contained across individual images in an angiographic sequence. Thus, our MIL model leverages a deep learning architecture demonstrating state-of-the-art performance on WSI pathology classification [17].

Paralleling the mode model approach, the ResNet50 CNN, pre-trained on ImageNet, serves as the classification backbone for feature extraction. A transformer encoder is attached to the end of that backbone after average pooling to account for dependencies in features identified in individual images within a sequence. The output of the transformer is fed into an attention mechanism that computes weights using a multilayer perceptron network. These aggregated features are subsequently passed to a linear classifier function to perform multiclass classification (Fig. 3B).

The MIL model architecture implemented is permutation-invariant by design. The temporal relationship between images within the sequence is not captured in this architecture as the inputs to both the transformer encoder and attention mechanism do not contain information on the sequential position of the image. This study’s focus on accurate sequence-level classification, rather than modeling temporal dependencies across images, aligns with the capabilities of the MIL architecture.

The training approach, data augmentation strategies, and hyperparameters are described in the Supplemental Materials.

### Model Evaluation and Statistical Analysis

Diagnostic performance for each model was evaluated on held-out test data. Statistical analyses were conducted using the scikit-learn 1.3.2 [18] and statsmodels 0.14.1 [19] python packages.

The primary performance endpoint of the study is the overall multiclass classification accuracy of the Mode models, trained with the full (D_Full_) or “key” image only (D_Key_) datasets, and the MIL model, trained with the full dataset. To assess the impact of excluding non- “key” images during training and testing, each Mode model is evaluated using both D_Full_ and D_Key_ datasets. The multiclass classification accuracies are compared for statistically significant differences using McNemar’s test [20]. *P*-values less than 0.05 were considered statistically significant. Frequency-weighted precision, recall, and F1 measures for the overall test data and precision, recall, and F1 measures per class are additionally studied. Confidence intervals (CI) for the multi-class measures were computed by bootstrapping the test data over 1000 iterations.

For evaluation of the MIL algorithm attention weights, images corresponding to the algorithmically generated top-5 attention weights were compared with manually labeled “key” images for overlap. The percent overlap was quantified as the number of images in common between the top-5 weighted images of the MIL algorithm and the “key” images divided by the minimum of 5 or the total number of key images.

## Results

### Dataset Characteristics

A total of 276 angiographically guided procedures from 205 unique patients were included in the final data for model development and evaluation. These procedures resulted in 819 unique angiographic sequences comprising 21,129 individual DSA images. These sequences accounted for 22.6% of the total DSA sequences obtained during the 276 procedures. The interobserver agreement kappa co-efficient for angiographic location class was 0.989. The kappa co-efficient for labeling both initial and final “key” images was 0.629. A total of eight patients were excluded from the final dataset due to transplant renal artery anastomoses.

The random 70/15/15 data partition resulted in 579 training, 121 validation, and 119 testing sequences, each containing non-overlapping patients (Fig. 2). Patient characteristics and anatomical locations for the training, validation, and testing datasets are summarized in Table 1.

**Table 1:** Characteristics of the data

	**Training dataset**	**Validation dataset**	**Testing dataset**
Total patients	150	33	32
Male	90	22	19
Female	60	11	13
Age (y), Std	57 ± 14	58 ± 16	58 ± 14
DSA sequences	579	121	119
DSA images	14942	3363	2824
“Key” DSA images	5469	1507	1063
DSA sequences by location			
Aorta	81	15	17
Celiac trunk	166	36	38
SMA	88	20	19
IMA	36	8	8
EIA, right	175	35	30
EIA, left	33	7	7

### Information Density

**Fig. 4:**
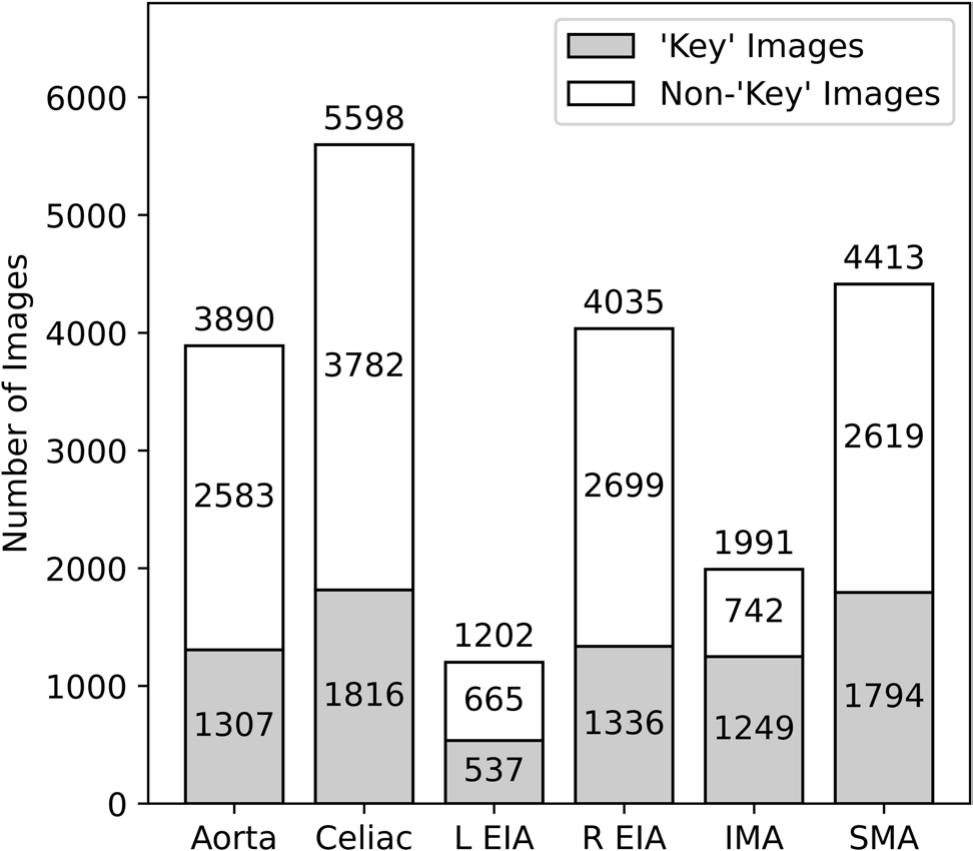
Information density. Diagnostic “key” images represent images demonstrating opacification of the index artery and the first downstream bifurcation such that the location may be inferred. The remainder of the images are labeled non-“key.” The images are grouped by label class. R, right; L, left; EIA, external iliac artery; IMA, inferior mesenteric artery; SMA, superior mesenteric artery

“Key” images comprised a minority of the total image data. The number of manually labeled diagnostic “key” images was 8039 representing 38.0% of the total 21,129 images in the dataset. The percentage of diagnostic images for each anatomic label varied from 33.1% for DSA sequences of the right EIA to 62.7% for sequences of the IMA. The per-class number of “key” and non- “key” images is presented in Fig. 4.

### Model Performance

The overall performance measures, including accuracy, precision, recall, and F1-score, with confidence intervals, are presented in Table 2. Statistically significant differences in the primary accuracy endpoint as assessed by McNemar’s test are explicitly highlighted. Performance measures across each anatomic location are presented in the Supplementary Materials.

**Table 2.**
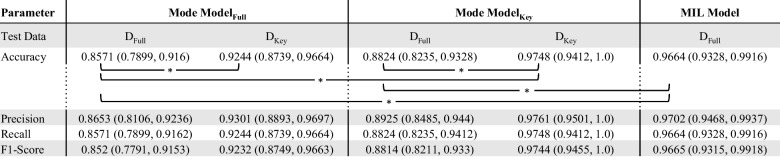
Overall performance measures for the Mode and the MIL models. Mode Model_Full_ and MIL Model are each trained using the full dataset (D_Full_). Mode Model_Full_ was trained using the “key” image only (D_Key_). The performance of each Mode model is evaluated seperately using the D_Full _and D_Key_ datasets. 95% confidence intervals in parentheses. Statistically significant differences in the accuracies are labeled with an asterisk

The multiclass classification results for the Mode Model_Full_ show an overall accuracy of 0.8571 (95% CI 0.7983–0.916) and 0.9244 (95% CI 0.8739–0.9664) evaluated on the full dataset, D_Full_, and the “key” image dataset, D_Key_, respectively. The overall accuracy of the Mode Model_Key_ (trained and validated using D_Key_ data) was 0.8824 (95% CI 0.8235–0.9328) and 0.9748 (95% CI 0.9412–1) when evaluated on the D_Full_ and D_Key_ testing datasets, respectively. The MIL model classification performance on the D_Full_ dataset demonstrated an overall accuracy of 0.9664 (95% CI 0.9328–0.9916).

Restricting the training and/or testing data to the “key” images only for the Mode models resulted in mixed statistical significance for accuracy improvement as evaluated by McNemar’s test. The Mode Model_Key_ accuracy evaluated with the D_Key_ dataset demonstrated statistically significant improvement over the Mode Model_Full_ evaluated using the D_Full_ dataset (*p* < < 0.001) and as compared to its own performance when evaluated with the D_Full_ dataset (*p* < 0.001). The Mode Model_Full_ evaluated using the D_Key_ dataset demonstrated statistically significant improvement (*p* = 0.021) as compared to its own performance when evaluated using the D_Full_ dataset. Conversely, Mode Model_Full_ evaluated using the D_Key_ dataset did not demonstrate statistically significant improvement (*p* = 0.302) as compared to the Mode Model_Key_ evaluated using the D_Full_ dataset.

Evaluating with the D_Full_ data, the MIL model demonstrated statistically significant improvement across all of the performance measures as compared to the Mode Model_Full_ as evidenced by the non-overlapping confidence intervals (Table 2). Comparing the model performance by McNemar’s, the MIL model demonstrated statistically significant improvement in accuracy as compared to the Mode Model_Full_ (*p* < < 0.001) and Mode Model_Key_ (*p* = 0.006), evaluated using the D_Full_ dataset. No statistically significant difference in accuracy was observed between the MIL model and the Mode models when evaluating these models using the D_Key_ dataset.

**Fig. 5:**
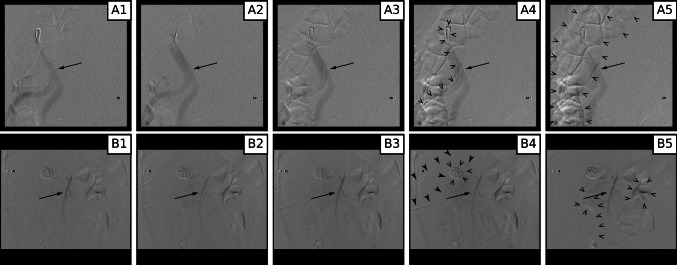
“Key” images from DSA sequences of the aorta (**A**) and superior mesenteric artery (**B**) misclassified by the MIL algorithm. Variable contrast within the vessels is highlighted by arrows in all frames (A and B). Multiple artifacts are created by motion including the angiographic catheter (open arrowheads, frame A4), bowel (open arrowheads, frame A5), internal hardware (open arrowheads, frame B4), overlying support hardware (solid arrowheads, frame B4), and renal contrast excretion (open arrowheads, frame B5)

DSA sequences misclassified by the algorithms demonstrate the challenges of DSA interpretation in the abdomen and pelvis. Diagnostic “key” images from two sequences misclassified by the MIL model are depicted in Fig. 5 with artifacts highlighted. Artifacts related to motion independent of the contrast transit through the vessels degrade the image quality and reduce the contrast between the opacified vessel and background. Additionally, contrast opacification within the arteries through the sequence of diagnostic images is substantially variable in density and geographic extension.

**Fig. 6:**
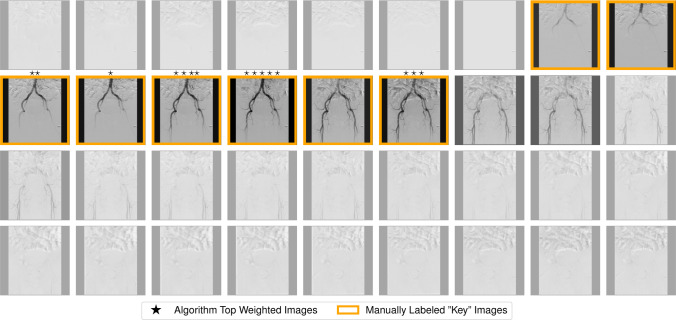
Comparison of manually labeled “key” images to weights of the MIL model attention mechanism. The opacity of each image in the DSA sequence of the aorta varies directly with the MIL algorithmically generated weights. Images with stars above represent the top five weighted images by the MIL model, with the star count correlating to the assigned weight. The manually labeled “key” images are framed in gold

The attention mechanism of the MIL model selected weights with high congruency to the “key” images. The average overlap between the algorithm-selected and manually-selected “key” images was 92.5%. At least one match between the “key” images and the algorithmically selected top-5 images occurred in 98.3% of the sequences. An illustrative example, Fig. 6, depicts a DSA sequence of the aorta labeled with both the “key” images and the attention weights assigned by the MIL model.

## Discussion

This study assessed the capability of deep learning algorithms to predict anatomical location in abdominopelvic DSA. One of the key challenges addressed is the ephemerality of the relevant findings in DSA sequence, as the sequences regularly capture substantial data before the contrast agent has reached the targeted anatomy and after it has dissipated. Thus, the deep learning models need mechanisms for identifying the important or diagnostic frames.

Diagnostic frame identification in an angiographic image sequence has been explored previously in unsubtracted coronary angiography through classical image filter and deep learning methodologies. Syeda et al. [21] employed classical image filter methods, including a Frangi filter [22], for identifying key frames with results demonstrating key-frame overlap to a manually selected frame in 90% of the approximately 210 cases. Moon et al. [23] use morphological dilation and erosion operations in combination with a Frangi filter to identify a single key frame with an average distance of 1.7 frames from a manually selected frame in 452 angiography sequences. Zhou et al. [24] demonstrate a supervised deep learning method for key frame extraction with a reported fourfold validation accuracy of 89% in a test set of 21 sequences.

In the data presented, less than half of the total images contained information deemed to be diagnostic during human interpretation with subject domain experts. The minority of image frames containing diagnostic data results in a sparsity of information for anatomic localization in the DSA sequences.

The information sparsity problem was solved, separately, through manual labeling and an attention mechanism. In developing the D_Key_ dataset and subsequently the Mode Model_Key_, a time-intensive labeling approach was advanced and evaluated. By identifying and utilizing only the diagnostic “key” images through manual labeling, favorable classification performance was achieved with significant improvement from an approach utilizing all image data. From a baseline of the entire dataset including “key” and non- “key” images, segmenting the “key” images for training, validation, and testing resulted in a statistically significant improvement of the overall classification accuracy by 13.7%.

This classification improvement was achieved at the significant cost of manual labeling of the “key” frames. The frame identification task added between 10 and 30 s per DSA. This accounted for an estimated minimum additional 10 h of domain expert labeling time with additional time required for review and adjudication of labeling discrepancies. This cost may stand as a substantial barrier to scalability of a manual “key” frame identification approach to larger and more diverse datasets. Notably, the performance improvement gains were significant when limiting the testing data with incremental non-significant improvement gained at the model development phase. Thus, optimal performance of algorithms for accurate localization using manual “key” frame identification would be limited to retrospective data analyses.

By deploying a MIL model with an attention mechanism, both the anatomic location and the diagnostic “key” image frames can be learned without the requirement for manual labeling of individual images. The attention mechanism selected images with high fidelity and close proximity to the manually identified “key” image frames as evidenced by the overlap performance. The MIL algorithm achieved comparable classification accuracy to the Mode Model_Key_, trained and tested with a manually filtered data rich “key” image set.

The misclassified angiographic sequences highlight the challenges of DSA interpretation in the abdomen and pelvis. Coronary angiography, which often does not require digital subtraction due to the relatively high image contrast and X-ray photon penetration, is unencumbered by the subtraction artifacts. In neuroangiography where digital subtraction is often required due to the density of the skull, unintentional motion may be more easily reduced by physical restraint. The data presented in this study consists of angiographic sequences obtained in routine clinical practice at a tertiary care medical center without exclusion of data with motion artifact, suboptimal contrast administration, and non-standard imaging parameters. These data thus reflect the imaging quality and artifacts with which automatic interpretation will need to function.

Anatomic classification algorithms for DSA have a multitude of immediate and future clinical applications. Location classification algorithms may be applied to automated assistance of report generation, coding, and billing. As interpretation of angiography is predicated on understanding the flow dynamics unique to a particular vascular territory, these algorithms may serve as a critical component of a fully automated DSA interpretation system.

The study design has several limitations to generalizability. The data are acquired from a single institution with imaging equipment from a single vendor. The retrospective nature of the data acquisition may produce bias with respect to the spectrum of vascular anatomy and pathology. Cases of post-surgical vascular anatomy, such as renal transplant with a donor artery anastomosed to the native external iliac artery, were explicitly excluded. Data used for the model development and evaluation were relatively small, constrained by the time required for manual labeling. While transfer learning techniques improved model performance, a larger and more balanced dataset could potentially enhance training and testing results. Additionally, the classification algorithm was limited to major first order anatomic structures of the abdomen and pelvis represented with relatively minor class imbalance. This excluded a substantial number of the total DSA sequences obtained during the procedures. As the goal of the study was to explore feasibility and performance of the methods for anatomic classification, the label set was limited by design to maximize the utility of manual labeling efforts and allow statistically significant comparisons of the multiclass performance. Expanding the classification to additional first and second order vascular structures may alter the model performance with investigation into this effect an objective for future study.

In summary, this study demonstrates the effectiveness of deep learning algorithms for anatomical localization using DSA images. Information sparsity in the DSA data was addressed through manual labeling and separately through an attention mechanism that automates identification of information. The algorithms may provide an initial critical step toward automatic interpretation, semi-automated report generation, and physician decision support in the angiography suite.

## Supplementary Information

Below is the link to the electronic supplementary material.

Supplementary file1 (PDF 222 KB)

## Data Availability

Data generated or analyzed during the study are available from the corresponding author by request. Data includes the de-identified digital subtraction angiography images, the sequence level anatomic labels, and the image level “key” and non-“key” labels. The underlying code for this study is currently available in the GitHub repository DSA Classification and may be accessed via the link https://github.com/regoldman/DSA_Classification.

## Declarations

### Standards of Reporting

This article has been developed in compliance with the Checklist for Artificial Intelligence in Medical Imaging ^25^.
