# Supplementary material for: Deep Learning Models for Automatic Classification of Anatomic Location in Abdominopelvic Digital Subtraction Angiography
Source: J Imaging Inform Med. 2025 Jan 9;38(5):2831–40. doi: 10.1007/s10278-024-01351-z (PMC12572449; doi:10.1007/s10278-024-01351-z)
Supplement: Supplementary file 1 — Supplementary file1 (PDF 222 KB) [file 10278_2024_1351_MOESM1_ESM.pdf]

## SUPPLEMENTARY MATERIAL

### Mode Model Training

The ResNet convolutional neural network (CNN) for each individual model was pretrained on ImageNet[1]. The CNNs were subsequently fine-tuned using the training sets, $D_{Full}$ or $D_{Key}$, respectively. Hyperparameter optimization was performed using the validation set. The models were each trained for 50 epochs using the Adam optimizer[2] with a learning rate of 1e-5, a cross entropy loss function, and a batch size of 100. Schedulers for optimization of the learning rate were evaluated without a substantial benefit in performance. Data augmentation, including linear contrast scaling, magnification and minification, and image rotation, was performed in-line on the input data during model training.

### MIL Model Training

The ResNet backbone utilized weights from a model pretrained on ImageNet. The CNN backbone was fine-tuned, and the transformer encoder component was trained, using the training data of Dataset $D_{Full}$, Figure 2. As the MIL model accepts image sequences of fixed length, for sequences with less than 50 images, blank images were padded to the end of the sequence to reach the desired 50 image frame length. The model was trained with a cross-entropy loss function for 50 epochs using the Adam optimizer. A cosine annealing learning rate scheduler adaptively adjusted the learning rate during training with initial rates of 2e-5 for the CNN parameters and 3e-5 for the transformer parameters. Hyperparameter optimization was performed using the validation set. Data augmentation, including linear contrast scaling, elastic deformation, affine rotation, and the addition of Gaussian noise, was performed in-line on the input data during model training.

### Automated Random Search for Data Split

For appropriate model development and evaluation, data must be partitioned into training, validation, and testing subsets containing non-overlapping patients as a minimum requirement for testing validity[3]. A split with the most even class balance across the training, validation, and testing subsets was chosen through a

search of random permutations of splits created at the patient level. The search method used the python function Pandas.DataFrame.sample with a random state to create the subsets and a weighted error to identify deviations from the intended 70/15/15 split for individual DSA sequences across all classes.

The patients were first split into two subsets, Train (70% fraction) and Val-Test (30% fraction), using a unique random state, i. The Val-Test patient subset was then split into two subsets Validation (50% fraction of the Val-Test representing 15% fraction of the total data) and Testing (50% fraction of the Val-Test representing 15% fraction of the total data) using a unique random state, j.

With this split complete, the percentages of DSA sequences for each anatomic location class were determined. By tabulating the total number of sequences labeled with a location class (abdominal aorta, celiac trunk, SMA, IMA, right EIA, and left EIA) in a subset (training, validation, and testing) and dividing by the total number of sequences for that class across all data, a percentage for each class in each data subset was calculated. A weighted error per class was calculated as the difference between the intended and actual percentages for each location class and each subset as follows:

$$weighted\_error[k] = train\_perc\_diff[k] + val\_perc\_diff[k] * 2 + test\_perc\_diff[k] * 2$$

where k is the individual location classes (abdominal aorta, celiac trunk, SMA, IMA, right EIA, and left EIA), and *train_perc_diff*, *val_perc_diff*, *tes_perc_diff* are the absolute values of the percentage differences between the intended and actual split for the training, validation, and testing subsets respectively.

Finally, an overall weighted error was calculated as the sum of the individual class weighted error. The data split algorithm was performed using 9e6 unique random state pairs (i, j). The data split with the lowest overall weighted error was selected.

**Model Performance Measures Across Each Anatomic Location**

| Anatomic Location | True Positive | False Negative | False Positive | True Negative | Precision (%) | Recall (%) | F1 (%) |
|---|---|---|---|---|---|---|---|
| aorta | 10 | 7 | 3 | 99 | 76.92 | 58.82 | 66.67 |
| celiac trunk | 36 | 2 | 4 | 77 | 90 | 94.74 | 92.31 |
| EIA, left | 6 | 1 | 1 | 111 | 85.71 | 85.71 | 85.71 |
| EIA, right | 30 | 0 | 1 | 88 | 96.77 | 100 | 98.36 |
| IMA | 4 | 4 | 0 | 111 | 100 | 50 | 66.67 |
| SMA | 16 | 3 | 8 | 92 | 66.67 | 84.21 | 74.42 |

*Table S1: Per class performance of the Mode Model$_{Full}$, Tested on All Image Data (Dataset $D_{Full}$). Abbreviations: EIA: external iliac artery, IMA: inferior mesenteric artery, SMA: superior mesenteric artery.*

| Anatomic Location | True Positive | False Negative | False Positive | True Negative | Precision (%) | Recall (%) | F1 (%) |
|---|---|---|---|---|---|---|---|
| aorta | 16 | 1 | 0 | 102 | 100 | 94.12 | 96.97 |
| celiac trunk | 36 | 2 | 2 | 79 | 94.74 | 94.74 | 94.74 |
| EIA, left | 7 | 0 | 2 | 110 | 77.78 | 100 | 87.5 |
| EIA, right | 30 | 0 | 1 | 88 | 96.77 | 100 | 98.36 |
| IMA | 5 | 3 | 0 | 111 | 100 | 62.5 | 76.92 |
| SMA | 16 | 3 | 4 | 96 | 80 | 84.21 | 82.05 |

*Table S2: Per class performance of the Mode Model$_{Full}$, Tested on "Key" Image Data (Dataset $D_{Key}$). Abbreviations: EIA: external iliac artery, IMA: inferior mesenteric artery, SMA: superior mesenteric artery*

| Anatomic Location | True Positive | False Negative | False Positive | True Negative | Precision (%) | Recall (%) | F1 (%) |
|---|---|---|---|---|---|---|---|
| aorta | 12 | 5 | 0 | 102 | 100 | 70.59 | 82.76 |
| celiac trunk | 37 | 1 | 8 | 73 | 82.22 | 97.37 | 89.16 |
| EIA, left | 6 | 1 | 0 | 112 | 100 | 85.71 | 92.31 |
| EIA, right | 28 | 2 | 1 | 88 | 96.55 | 93.33 | 94.92 |
| IMA | 7 | 1 | 1 | 110 | 87.5 | 87.5 | 87.5 |
| SMA | 15 | 4 | 4 | 96 | 78.95 | 78.95 | 78.95 |

*Table S3: Per class performance of the Mode Model$_{Key}$ Tested on All Image Data (Dataset D$_{Full}$).*

*Abbreviations: EIA: external iliac artery, IMA: inferior mesenteric artery, SMA: superior mesenteric artery*

| Anatomic Location | True Positive | False Negative | False Positive | True Negative | Precision (%) | Recall (%) | F1 (%) |
|---|---|---|---|---|---|---|---|
| aorta | 17 | 0 | 0 | 102 | 100 | 100 | 100 |
| celiac trunk | 38 | 0 | 1 | 80 | 97.44 | 100 | 98.7 |
| EIA, left | 6 | 1 | 0 | 112 | 100 | 85.71 | 92.31 |
| EIA, right | 30 | 0 | 2 | 87 | 93.75 | 100 | 96.77 |
| IMA | 7 | 1 | 0 | 111 | 100 | 87.5 | 93.33 |
| SMA | 18 | 1 | 0 | 100 | 100 | 94.74 | 97.3 |

*Table S4: Per class performance of the Mode Model$_{Key}$ Tested on "Key" Image Data (Dataset D$_{Key}$).*

*Abbreviations: EIA: external iliac artery, IMA: inferior mesenteric artery, SMA: superior mesenteric artery.*

| Anatomic Location | True Positive | False Negative | False Positive | True Negative | Precision (%) | Recall (%) | F1 (%) |
|---|---|---|---|---|---|---|---|
| aorta | 16 | 1 | 0 | 102 | 100 | 94.12 | 96.97 |
| celiac trunk | 38 | 0 | 1 | 80 | 97.44 | 100 | 98.7 |
| EIA, left | 6 | 1 | 0 | 112 | 100 | 85.71 | 92.31 |
| EIA, right | 30 | 0 | 1 | 88 | 96.77 | 100 | 98.36 |
| IMA | 8 | 0 | 2 | 109 | 80 | 100 | 88.89 |
| SMA | 17 | 2 | 0 | 100 | 100 | 89.47 | 94.44 |

*Table S5: Per class performance of the MIL Model Developed and Tested on All Image Data (Dataset D$_{Full}$).*

*Abbreviations: EIA: external iliac artery, IMA: inferior mesenteric artery, SMA: superior mesenteric artery.*

## REFERENCES


1. Russakovsky, O. *et al.* ImageNet Large Scale Visual Recognition Challenge. *Int. J. Comput. Vis.* **115**, (2015).
2. Kingma, D. P. & Ba, J. L. Adam: A method for stochastic optimization. in *3rd International Conference on Learning Representations, ICLR 2015 - Conference Track Proceedings* (2015).
3. Mongan, J., Moy, L. & Kahn, C. E. Checklist for Artificial Intelligence in Medical Imaging (CLAIM): A Guide for Authors and Reviewers. *Radiol. Artif. Intell.* **2**, e200029 (2020).
